# Influence of perceived harm due to substance use on the relationships between positive psychotic experiences and suicidal experiences in people with non-affective psychosis

**DOI:** 10.1017/S0033291724001764

**Published:** 2024-09

**Authors:** Patricia Gooding, Kamelia Harris, Paula Duxbury, Daniel Pratt, Charlotte Huggett, Richard Emsley, Yvonne Awenat, Gillian Haddock

**Affiliations:** 1 Division of Psychology and Mental Health, Faculty of Biology, Medicine and Health, School of Health Sciences, University of Manchester, Manchester, UK; 2 Manchester Academic Health Science Centre, MAHSC, University of Manchester, Manchester, UK; 3 Greater Manchester Mental Health NHS Foundation Trust, Suicide, Risk and Safety Research Unit, Manchester, UK; 4 Department of Biostatistics and Health Informatics, Institute of Psychiatry, Psychology & Neuroscience, Kings College London, London, UK

**Keywords:** active suicidal thoughts, alcohol use, auditory hallucinations, delusions, drug use, passive suicidal thoughts, psychosis, schizophrenia, substance use, suicide

## Abstract

**Background:**

The ways in which perceived harm due to substance use affects relationships between psychotic and suicidal experiences are poorly understood. The goal of the current study was to redress this gap by investigating the moderating effects of harm due to substance use on pathways involving positive psychotic symptoms, the perceived cognitive-emotional sequelae of those symptoms, and suicidal ideation.

**Method:**

The design was cross-sectional. Mediation and moderated mediation pathways were tested. The predictor was severity of positive psychotic symptoms. Cognitive interpretative and emotional characteristics of both auditory hallucinations and delusions were mediators. Suicidal ideation was the outcome variable. General symptoms associated with severe mental health problems were statistically controlled for.

**Results:**

There was evidence of an indirect pathway between positive psychotic symptom severity and suicidal ideation via cognitive interpretation and emotional characteristics of both auditory hallucinations and delusions. Harm due to drug use, but not alcohol use, moderated the indirect pathway involving delusions such that it was most prominent when harm due to drug use was at medium-to-high levels. The components of suicidal ideation that were most strongly affected by this moderated indirect pathway were active intent, passive desire, and lack of deterrents.

**Conclusions:**

From both scientific and therapy development perspectives, it is important to understand the complex interplay between, not only the presence of auditory hallucinations and delusions, but the ensuing cognitive and emotional consequences of those experiences which, when combined with harm associated with substance use, in particular drug use, can escalate suicidal thoughts and acts.

## Introduction

Suicidal experiences including thoughts, urges, and plans are often multi-componential, accompanied by extreme psychological pain and distress, and, therefore, should be an urgent clinical target (Harris, Gooding, Peters, & Haddock, 2020; Tarrier et al., 2013; Tarrier et al., 2014). They are also frequent precursors to suicide attempts and deaths (Chapman et al., 2015). Suicidal thoughts/acts are substantially elevated in people with mental health problems including those with a diagnosis of bipolar disorder, personality disorder, and schizophrenia (Chesney, Goodwin, & Fazel, 2014; Correll et al., 2022). A recent meta-analysis reported that suicide deaths were 4.5 times more likely for people with schizophrenia compared to the general population (Olfson et al., 2021).

The high prevalence of suicide fatalities in those with problematic substance use involving alcohol and drugs has also been widely documented (Armoon et al., 2021; Correll et al., 2022; Hunt, Large, Cleary, Lai, & Saunders, 2018; Poorolajal, Haghtalab, Farhadi, & Darvishi, 2016). Using drugs and/or alcohol to levels considered harmful is highly prevalent in people with a diagnosis of schizophrenia (Preti, Meneghelli, Pisano, Cocchi, & Programma, Team, 2009). Indeed, a recent meta-analysis reported that 41.7% of individuals with schizophrenia had co-morbid substance use problems (Hunt et al., 2018). This type of comorbidity substantially elevates the frequency and severity of suicidal thoughts, attempts, and fatalities (Lähteenvuo et al., 2021; Large, Mullin, Gupta, Harris, & Nielssen, 2014; Olfson et al., 2021).

Evidence is growing which documents relationships between positive psychotic symptoms (e.g. hallucinations, delusions, and paranoid thoughts and feelings) and suicidal thoughts and attempts (Bolton, Gooding, Kapur, Barrowclough, & Tarrier, 2007; Bornheimer, 2016; de Cates et al., 2021; Huang, Fox, Ribeiro, & Franklin, 2018). For example, in a recent household survey, persecutory thoughts mediated the relationship between auditory hallucinations and suicidal experiences cross-sectionally. Furthermore, persecutory ideas and auditory hallucinations fed into suicidal thoughts over time (de Cates et al., 2021). However, it is important to consider not only the influence of psychotic symptoms themselves on suicidal experiences but also the impact of the severity of the cognitive and emotional sequelae of those symptoms on suicidal thoughts/acts (Chawla, Deep, Khandelwal, & Garg, 2019; Haddock, McCarron, Tarrier, & Faragher, 1999; Schneider, Jelinek, Lincoln, & Moritz, 2011). This distinction is especially pertinent because frequently used psychiatrically derived measures of positive psychotic symptoms often group together different components of psychotic symptoms, specifically, hallucinations, delusions, excitement, grandiosity, conceptual disorganization, suspiciousness/persecution, and hostility, and do not assess the cognitive and emotional impact on an individual of those different symptoms (Kay, Fiszbein, & Opler, 1987).

The negative effects of living with auditory hallucinations and delusions have been differentiated in terms of their perceived cognitive and emotional properties (Haddock et al., 1999). For instance, both hallucinations and delusions can feel controlling, hijacking, or all-consuming, and result in individuals being preoccupied with discerning the origin and meaning of those experiences. Hallucinatory voices often convey negative content (e.g. being a failure and/or being worthless) as can delusional beliefs (e.g. being persecuted) and often seem unrelenting and exceptionally frightening (Upthegrove, 2018). Hence, both delusions and hallucinations frequently result in severe levels of emotional distress. Studies which have examined perceptions of these cognitive and emotional characteristics of auditory hallucinations and delusional experiences in relation to suicide are limited. That said, two recent studies did report associations between the emotional impact of auditory hallucinations on suicidal thoughts in people with a diagnosis of schizophrenia (Chong et al., 2020; Yin et al., 2023). This work represents a promising direction but is in its infancy.

There are three clear gaps in the literature. First, work has not yet investigated mediated pathways between the presence of positive psychotic symptoms in general, the severity of the cognitive interpretations and emotional impact of those symptom, particularly auditory hallucinations and delusions, and resultant suicidal thoughts and behaviors. Second, the extent to which harmful levels of substance use impact such mechanistic pathways to suicidal experiences have not been investigated. Third, suicidal thoughts are multi-faceted and encompass numerous components, such as intensity, planning, active desire, passive attempts, and lack of deterrents (Beck & Steer, 1991). It is important from clinical and scientific perspectives to determine the extent to which these components of suicide experiences may be differentially affected by pathways involving the cognitive interpretation and emotional consequences of auditory hallucinations and delusions, and substance use.

**Figure 1.**
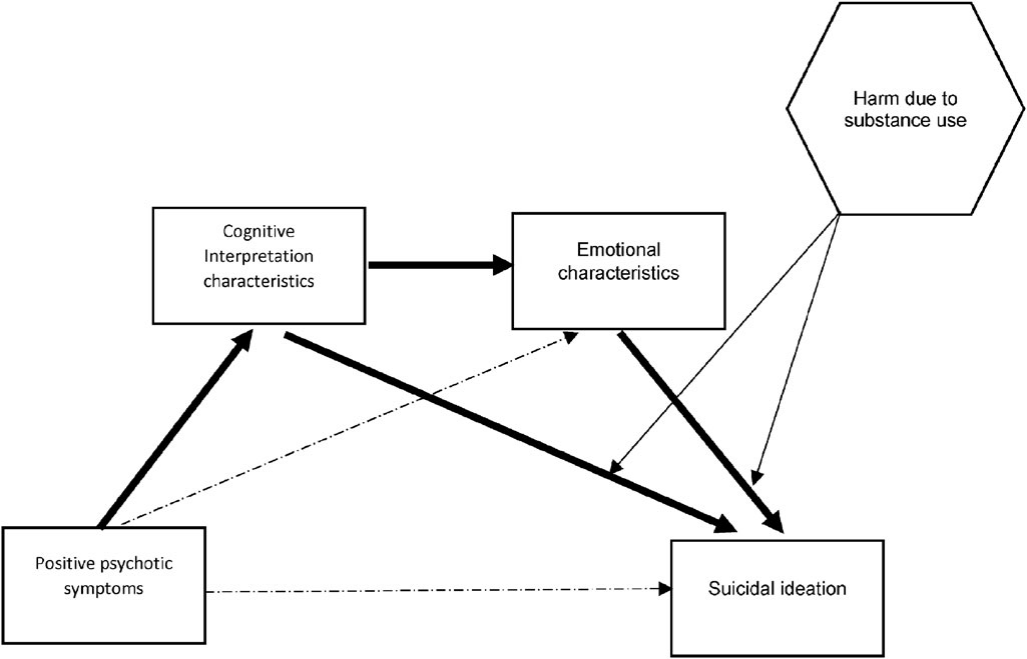
Postulated moderated mediated pathway from the presence of positive psychotic symptoms to suicidal ideation via two mediators of cognitive interpretation and emotional characteristics of delusions and auditory hallucinations. The moderator is perceived harm due to substance use. The predicted mediated pathways are shown as solid lines.

Hence, this study addressed the following three research questions: the first was, to what extent did the perceived severity of the cognitive and/or emotional characteristics of auditory hallucinations and delusions act as mediators between the presence of positive psychotic symptoms in general and suicidal ideation? For both auditory hallucinations and delusions, an exploratory prediction was that cognitive interpretations of symptoms (e.g. disruption to life) would feed into negative emotional characteristics of, and reactions to, those symptoms (e.g. distress). The perceived cognitive interpretations and negative emotional characteristics of symptoms were both tentatively expected to be linked to suicidal thoughts (see Fig. 1). Therefore, a mediated pathway was explored in which the presence of positive psychotic symptoms in general was associated with suicidal thoughts indirectly via the severity of the perceived cognitive and emotional characteristics of auditory hallucinations and delusions. The second research question was to what extent did self-identified harm due to drug or alcohol use moderated mediation pathways from positive psychotic symptoms to suicidal ideation? It was expected that these pathways would be strongest with high levels of perceived harm due to substance use. The third research question was which of the different components of suicidal ideation were most affected by moderated mediated pathways?

## Method

### Design

The design was cross-sectional and correlational, using baseline assessments from the Cognitive AppRoaches to coMbatting Suicidality (CARMS) project which recruited 292 participants (Gooding et al., 2020).

### Participants

Participants were 18 years or over; had a diagnosis relating to non-affective psychosis (ICD-10 criteria, F20–29) (World Health Organization, 2004); current suicidal experiences or had such experiences in the 3 months prior to recruitment. Participants were largely recruited from National Health Service community and hospital mental health teams (e.g. inpatient psychiatric wards, crisis teams, early intervention services, and home treatment teams) (Gooding et al., 2020). Diagnoses were based on case notes, the mental health care team of the participant, and when unclear were discussed with a practicing psychiatrist and academic. Participants were excluded if they (i) had dementia or an organic brain disorder, (ii) did not have sufficient English language abilities to complete assessments, (iii) were unable to provide informed consent, or (iv) were participating in another psychological treatment trial (Gooding et al., 2020). For the current study, only participants who self-reported some level of harm due to drug or alcohol use were included. This was because the research question was focused on the effects of self-identified harm due to substance use along a continuum, as opposed to a binary of harm *v.* no harm, for which a range of harm from small to large was optimal. The number of participants who reported some degree of harm due to substance use was 212.

### Measures

1. The Beck Scale for Suicidal Ideation (BSS) (Beck & Steer, 1991) is self-administered, contains 19 items (scored 0, 1, or 2) which measures suicidal thought severity. Two further items measure lifetime suicide attempts, and severity of the most recent attempt. The BSS has five components: intensity, active desire, plans, passive attempts, and lack of deterrents to suicide. The alpha reliability (Cronbach's alpha) was 0.98 for the current sample.
2. The Positive and Negative Syndrome Scale (PANSS) (Kay et al., 1987) is a semi-structured clinical interview evaluating positive psychotic symptoms (7 items), negative psychotic symptoms (7 items), and general symptoms (16 items). Only the positive and general symptom scales were used. Each item is scored from 1 to 7. Items P4 (excitement), N1 (blunted affect), G4 (tension), G5 (mannerisms and posturing), G7 (motor slowing), G11 (poor attention), G13 (disturbance of volition), and G14 (poor impulse control) were missing for 45 participants because they are scored using interviewer observation and the PANSS was carried out by telephone for a sub-sample of individuals due to UK COVID-19 lock-down restrictions. The inter-rater reliabilities for the research assistants conducting the PANSS was 0.87 for the positive psychotic symptom scale and 0.69 for the general symptom scale.
3. The Psychotic Symptom Rating Scale (PSYRATS) (Haddock et al., 1999) measures the severity of dimensions of auditory hallucinations (11 items) and delusions (6 items) with two scales. Items are scored 0–4 reflecting increasing severity. There are three components of the hallucinations scale which are perceptions of the emotional characteristics of the voices (4 items: amount and degree of negative content of voices, and amount and intensity of distress); the physical characteristics of the voices (4 items: frequency, duration, location, and loudness), and cognitive interpretation of the voices (4 items: amount and duration of preoccupation, conviction, disruption to life). The delusions scale has two sub-scales, namely, perceptions of the emotional characteristics of the beliefs (2 items: amount and intensity of distress), and cognitive interpretation of the beliefs (4 items: amount and duration of preoccupation, conviction, disruption to life). The PSYRATS is administered as part of a semi-structured clinical interview. The alpha reliability was 0.95 for the auditory hallucination scale and 0.92 for the delusions scale.
4. The Alcohol Use Disorders Test (AUDIT) (Day, Copello, & Hull, 2015) uses 10 items scored from 0 to 4 to measure self-perceived harm due to drinking alcohol. Potential harm can be categorized as low-risk, hazardous, harmful, and possible dependency. The alpha reliability was 0.86 for the current sample.
5. The Drug Abuse Screening Test (DAST) (Skinner, 1982) 20-item version, measures harm due to drug use. It is self-administered and records perceptions of harm due to drug use. Item scores are 0 or 1. The probability of harm due to using drugs can be categorized as low, intermediate, substantial, and severe. The alpha reliability was 0.89 for the current sample.

### Procedure

Measures were administered as part of the CARMS project (Gooding et al., 2020) by trained research assistants over as many sessions as required by participants.

### Statistical analyses

Only participants with scores on harm due to alcohol or drug use which were equal to or greater than 1 were included in the analyses. Hence, it was a requirement that all participants perceived that using drugs and/or alcohol had caused them some level of harm. Items which were not completed were not pro-rated but treated as missing items. The predictor variable was severity of positive psychotic symptoms (PANSS psychotic symptoms sub-scale). The outcome variable was severity of suicidal thoughts. The four mediator variables were cognitive interpretations and emotional characteristics of delusions, and cognitive interpretations and emotional characteristics of auditory hallucinations (PSYRATS). The two moderator variables were harm due to drug or alcohol use. The control variable was general psychiatric symptoms (PANSS general sub-scale). Associations between variables were analyzed with Spearman's rho correlation coefficients. Mediated pathways and moderated mediation pathways were assessed with linear regression models with mean-centered interaction terms between predictor and moderator variables. Significant moderation effects were probed with conditional effects on the outcome variable of the predictor variable at the mean, the mean + 1 standard deviation (s.d.), and the mean −1 s.d. of the moderator variable. For mediated analyses, direct and indirect effects were examined. Where there were significant moderated mediation effects, the indirect effect at three levels of the moderator variable were examined together with pairwise contrasts between the conditional indirect effects (Hayes, 2020). Bootstrapping with 5000 iterations was applied because data distributions were skewed. The type 1 error was 0.05 and results were reported with bootstrapped 95% confidence intervals (CIs). The mediated pathways were analyzed with Process software template 4 and the moderated mediation pathways with template 58 (Hayes, 2020) using SPSS version 25. Graphical visualization of significant moderation effects used the data generated by Process.

## Results

### Participant characteristics

Participants were aged 18–70. The majority (65.6%) had a diagnosis of schizophrenia; identified as being of white/Caucasian ethnicity; were single; lived alone; had no dependents; and were exempt from employment because of disability (see Table 1).

**Table 1.** Demographic characteristics of participants who perceived that they had some level of harm due to drugs and/or alcohol use (*N* = 212)

Age (years) (mean, [s.d.], range)	34.69, [13.17], 18–69.92	
Gender	Female	90
	Male	121
	Missing	1
Diagnosis	Schizophrenia	139
	Schizoaffective disorder	29
	Persistent delusional disorder	8
	Acute and transient psychotic disorder	4
	Schizotypal disorder	2
	Unspecified non-organic psychosis	25
	Other non-organic psychotic disorder	5
Ethnicity	White/Caucasian	195
	Asian	7
	Mixed/multiple ethnicities	4
	Black African/Caribbean	1
	Other	4
	Would rather not say	1
Relationship status	Single	149
	Married/living with a partner	30
	In a relationship (not cohabiting)	14
	Divorced	10
	Separated	5
	Widow/widower	2
	Missing	2
Living situation	Alone	76
	With parents	61
	With spouse/partner	32
	With other relatives (not parents)	16
	With friends and/or other people	15
	Supported accommodation	6
	Inpatient psychiatric ward	4
	Homeless	1
Dependents	Yes	51
	No	161
Employment status	Exempted through disability	74
	Unemployed	56
	Paid or self-employed	27
	Voluntary work	8
	Student	5
	Retired	5
	Housewife/househusband	1
	Missing	3

### Clinical characteristics of participants and correlation coefficients between clinical variables

Thirty-nine (18%) participants had never attempted suicide in their lifetime, 43 (20%) had attempted suicide once, and 125 (59%) had attempted to take their life more than once. One-hundred-and-twenty-two (73%) indicated that at their last suicide attempt their wish to die had been high. The median suicidal ideation score was 16 (mean = 15.87), with the maximum score being 35 out of a possible maximum of 38 (see Table 2).

**Table 2.** Descriptive statistics (mean [Mn], standard deviation [s.d.], range and sample size [*N*]) and Spearman rho correlation coefficients for predictor, mediator, moderator, control, and the outcome variables

	Mn, (s.d.), Range, *N*	2	3	4	5	5.1	5.2	6	6.1	6.2	7
1. Harm due to alcohol use (AUDIT)	9.72 (8.14) 1–34 *N* = 177	0.23** N = 159	0.04 *N* = 177	−0.02 *N* = 177	−0.11 *N* = 171	−0.14 *N* = 169	0.10 *N* = 166	−0.04 *N* = 171	−0.05 *N* = 171	−0.01 *N* = 172	−0.01 *N* = 174
2. Harm due to drug use (DAST)	5.11 (4.18) 1–19 *N* = 136		0.11 *N* = 136	−0.02 *N* = 136	0.01 *N* = 133	0.07 *N* = 130	0.11 *N* = 130	−0.01 *N* = 132	0.01 *N* = 132	0.004 *N* = 132	−0.06 *N* = 131
3. PANSS general psychiatric symptoms (*possible range 16–112*)	35.19 (7.57) 14–58 *N* = 212			0.61** N = 212	0.29** N = 206	0.23** N = 202	0.30** N = 201	0.41** N = 206	0.39** N = 206	0.34** N = 207	0.30** N = 207
4. PANSS positive psychotic symptoms (*possible range 7–49*)	17.19 (5.12) 6–31 *N* = 212				0.44** N = 206	0.37** N = 202	0.41** N = 201	0.67** N = 206	0.71** N = 201	0.47** N = 207	0.27** N = 207
5. PSYRATS total Auditory hallucinations (*possible range 0–44*)	21.51 (13.93) 0–41 *N* = 206					0.407**	0.374**	0.41** N = 201	0.38** N = 201	0.36** N = 202	0.37** N = 201
*5.1 PSYRATS cognitive characteristics* (*possible range 0–16*)	7.55 (4.78) 0–14 *N* = 202						0.74** N = 198	0.36** N = 198	0.34** N = 198	0.31** N = 199	0.29** N = 197
*5.2 PSYRATS emotional characteristics* (*possible range 0–16*)	8.40 (6.30) 0–16 *N* = 201							0.37* N = 197	0.34** N = 197	0.35*8 *N* = 198	0.34** N = 196
6. PSYRATS delusions (*possible range 0–24*)	14.06 (7.02) 0–24 *N* = 206								0.95** N = 206	0.84** N = 206	0.29** N = 201
*6.1 PSYRATS cognitive characteristics* (*possible range 0–16*)	8.93 (4.58) 0–16 *N* = 206									0.66** N = 206	0.24** N = 201
*6.2 PSYRATS emotional characteristics* (*possible range 0–8*)	5.16 (2.78) 0–8 *N* = 207										0.31** N = 202
7. Suicidal ideation (*possible range 0–38*)	15.87 (8.18) 0–35 *N* = 207										

Descriptive statistics and correlation coefficients involving drug use only include participants who scored 1 or more on the harm due to alcohol use measure (AUDIT). Similarly, descriptive statistics and correlation coefficients involving drug use only include participants who scored 1 or more on the measure of harm due to drug use (DAST).

**p < 0.01.

Mean scores for psychotic symptoms measured by the PANSS positive subscale were the highest for P1 (delusions: mean = 3.9, s.d. = 1.50), followed by P6 (suspiciousness and persecution: mean = 3.78, s.d. = 1.41), P3 (hallucinatory behaviors: mean = 3.66, s.d. = 1.56), P5 (grandiosity: mean = 1.87, s.d. = 1.43); P4 (excitement: mean = 1.59, s.d. = 0.91), P2 (conceptual disorganization: mean = 1.52, s.d. = 0.97); and P6 (hostility: mean = 1.21, s.d. = 0.61).

PANSS positive psychosis symptom scores were positively and significantly correlated with total PSYRATS and cognitive interpretations and emotional characteristics of delusions and auditory hallucinations (see Table 2). There were also positive and significant correlation coefficients between suicidal ideation severity and PANSS general scores, PANSS positive scores, total hallucination and delusion PSYRATS scores, and PSYRATS cognitive interpretation and emotional characteristics subscales for both delusions and hallucinations. There were no significant correlation coefficients between harm due to drug or alcohol use and suicidal ideation, PANSS positive psychotic symptoms, nor total PSYRATS and PSYRATS cognitive interpretation and emotional characteristics of hallucinations or delusions (see Table 2).

**Research Question 1: To what extent did the perceived severity of the PSYRATS cognitive interpretation and emotional characteristics of auditory hallucinations and delusions act as mediators between the presence of PANSS positive psychotic symptoms and suicidal ideation?**

**Figure 2.**
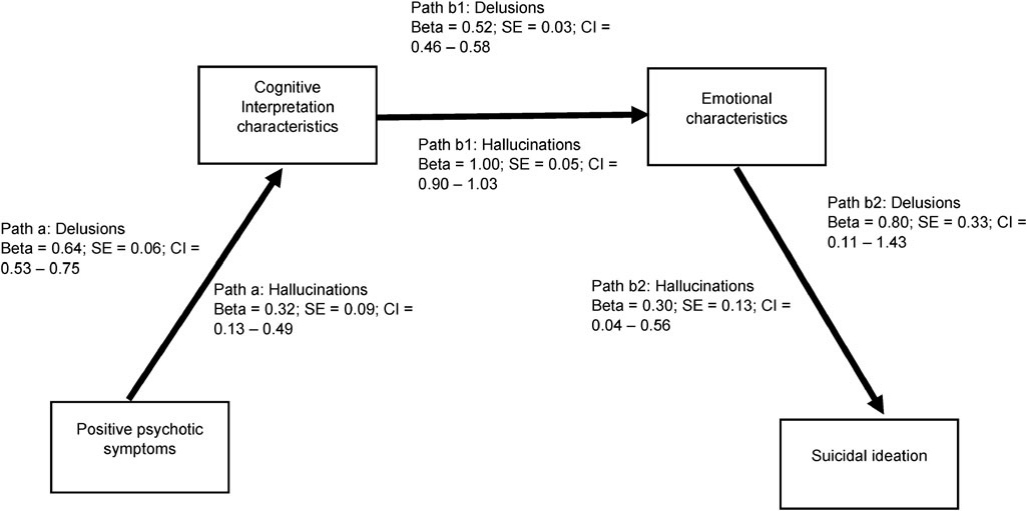
Significant mediation pathways from positive psychotic symptoms to suicidal ideation via two sequential mediators of cognitive interpretation characteristics and emotional characteristics of delusions (*N* = 201) and auditory hallucinations (*N* = 193) with general psychiatric symptoms as a control variable. This was the only significant indirect effect (a × b1 × b2) for both delusions (effect = 0.27, s.e. = 0.11, CI = 0.04–0.49) and hallucinations (effect = 0.06, s.e. = 0.04, CI = 0.006–0.12). The direct effect (path c′) from positive psychotic symptoms to suicidal ideation was not significant for delusions (effect = 0.06, s.e. = 0.17, CI = −0.28 to 0.40) nor for hallucinations (effect = 0.04, s.e. = 0.14, CI = −0.24 to 0.31).

As shown in Fig. 2, the hypothesized mediation pathway was partially supported. PANSS psychotic symptoms were associated with suicidal ideation indirectly via two sequential mediators of cognitive interpretation and emotional characteristics of delusions and auditory hallucinations as measured by the PSYRATS. For both delusions and auditory hallucinations, there were significant associations between (i) PANSS psychotic symptoms and cognitive interpretation characteristics (path a); (ii) cognitive interpretation characteristics and emotional characteristics (path b1); and emotional characteristics and suicidal ideation (path b2). This indirect path was the only one which was statistically significant. The predicted pathway from PANSS positive psychotic symptoms to suicidal ideation via cognitive interpretation characteristics did not reach significance. PANSS positive psychotic symptoms were not associated with suicidal ideation directly but were associated indirectly while statistically controlling for general psychiatric symptoms.

It was predicted that the mediator of PSYRATS cognitive interpretations would precede the mediator of emotional characteristics. However, as this study was cross-sectional, it was possible that emotional characteristics would precede cognitive interpretations. When this alternative pathway was tested, for delusions, no indirect effect was significant, and for auditory hallucinations only an indirect effect from positive symptoms to emotional characteristics to suicidal ideation was supported (effect = 0.15, s.e. = 0.09, CI =  0.01–0.36) with the alternative pathway in which emotional characteristics preceded cognitive interpretation characteristics failing to reach significance. This lends reassurance that the data best supported the model depicted in Fig. 2.

**Research Question 2: To what extent did harm due to drug or alcohol use moderate pathways from PANSS positive psychotic symptoms to suicidal ideation via the severity of the cognitive interpretation and emotional characteristics of delusions and auditory hallucinations as measured by the PSYRATS?**

To examine the effects of harm due to substance use, the pathways were broken down into three moderated mediation models and analyzed using Process model 58 (Hayes, 2020):

1. PANSS positive symptoms (predictor) →  PSYRATS cognitive interpretations (mediator) →  suicidal ideation (outcome)
2. PANSS positive symptoms (predictor) →  PSYRATS emotional characteristics (mediator) →  suicidal ideation (outcome)
3. PANSS positive symptoms (predictor) →  PSYRATS cognitive interpretations (mediator) →  PSYRATS emotional characteristics (outcome)

These three models were applied first, to delusions with harm due to drug use as a moderator; second to auditory hallucinations with harm due to drug use as a moderator; third, to delusions with harm due to alcohol use as a moderator; and fourth, to auditory hallucinations with harm due to alcohol use as a moderator. When harm due to drug use was a moderator, only participants who had some level of harm due to drug use (DAST score ≥1) were included; and when harm due to alcohol use was a moderator only participants who had some level of harm due to alcohol use (AUDIT score ≥1) were included.

*Harm due to drug use as a moderator with PSYRATS cognitive interpretation and emotional characteristics of delusions as mediators* (*N* *=* *127*)

PANSS positive symptoms of psychosis were significantly associated with both mediators of PSYRATS cognitive interpretations and emotional characteristics of delusions, and harm due to drug use did not affect (interact with) the strength of these associations. PANSS positive psychotic symptoms were not significantly associated with suicidal ideation (see Table 3). In contrast, the two mediators were significantly associated with suicidal ideation.

**Table 3.** Results of moderated mediation analyses with PANSS positive psychotic symptoms as the predictor variable and severity of suicidal ideation as the outcome variable, harm due to drug use (DAST) as the moderator, and PANSS general psychotic symptoms as the control variable

Model 1 (*N* = 127) mediator = delusions: cognitive interpretation characteristics; moderator = harm due to drug use			
	Outcome = delusions: cognitive interpretation characteristics		
	Coefficient	s.e.	95% CI
PANSS positive symptoms	0.68	0.08	0.52–0.83*
Harm due to drug use (DAST)	0.08	0.06	−0.05 to 0.20
Harm due to drug use × PANSS positive symptoms	−0.01	0.01	−0.03 to 0.02
PANSS general symptoms	−0.08	0.06	−0.19 to 0.04
	Outcome = suicidal ideation		
PANSS positive symptoms	−0.06	0.20	−0.44 to 0.35
PSYRATS cognitive interpretations	0.44	0.19	0.07–0.83*
Harm due to drug use (DAST)	−0.17	0.16	−0.51 to 0.12
Drug use × PSYRATS cognitive interpretations	0.11	0.04	0.03–0.20*
PANSS general symptoms	0.34	0.13	0.09–0.57*
Direct effect = −0.06, s.e. = 0.21, *t* = −0.30, *p* = 0.77, CI = −0.47 to 0.35			
*Indirect effect positive symptoms* *=>* *Cognitive interpretation characteristics* *=>* *Suicidal ideation at 3 levels of Harm due to drug use*			
	Effect	s.e.	95% CI
−1 s.d. = −4.06	0.005	0.18	−0.35 to 0.35
Mean 0	0.30	0.13	0.05 to 0.57*
+1 s.d. = 4.21	0.58	0.17	0.29 to 0.95*
*Pairwise contrasts between conditional indirect effects*			
Effect 1 *v*. effect 2	Contrast	s.e.	95% CI
Mean *v*. −1 s.d.	0.29	0.12	−0.08 to 0.58
+1 s.d. *v*. −1 s.d.	0.57	0.24	0.16–1.08*
+1 s.d. *v*. mean	0.30	0.12	0.07–0.54*
*Model 2* (*N* *=* *127*) *Mediator* *=* *delusions: emotional characteristics; moderator* *=* *harm due to drug use*			
	Coefficient	s.e.	95% CI
	Outcome = delusions: *emotional characteristics*		
PANSS positive symptoms	0.31	0.06	0.19–0.42*
Harm due to drug use (DAST)	0.03	0.05	−0.06 to 0.11
Harm due to drug use × PANSS positive symptoms	0.002	0.01	−0.02 to 0.02
PANSS general symptoms	−0.005	0.04	−0.08 to 0.07
	Outcome = suicidal ideation		
PANSS positive symptoms	0.03	0.18	−0.29 to 0.40
PSYRATS Emotional characteristics	0.55	0.26	0.03–1.05*
Harm due to drug use (DAST)	−0.14	0.15	−0.231 to 0.15
Drug use × PSYRATS Emotional characteristics	0.13	0.06	0.02–0.24*
PANSS general symptoms	0.31	0.13	0.06–0.54*
*Indirect effect Positive Symptoms* *=>* *Emotional characteristics* *=>* *Suicidal ideation at 3 levels of harm due to drug use*			
	Effect	s.e.	95% CI
−1 s.d. = −4.06	0.01	0.11	−0.22 to 0.22
Mean 0	0.17	0.08	0.01–0.34*
+1 s.d. = 4.21	0.34	0.13	0.10–0.62*
*Pairwise contrasts between conditional indirect effects*			
Effect 1 *v*. effect 2	Contrast	s.e.	95% CI
Mean *v*. −1 s.d.	0.16	0.08	0.02–0.32*
+1 s.d. *v*. −1 s.d.	0.33	0.16	0.03–0.66*
+1 s.d. *v*. mean	0.17	0.09	0.005–0.36*

In model 1, PSYRATS cognitive interpretation characteristics of delusions was the mediator. In model 2, PSYRATS emotional characteristics of delusions was the mediator. Significant effects have been highlighted with an asterisk (*).

**Figure 3.**
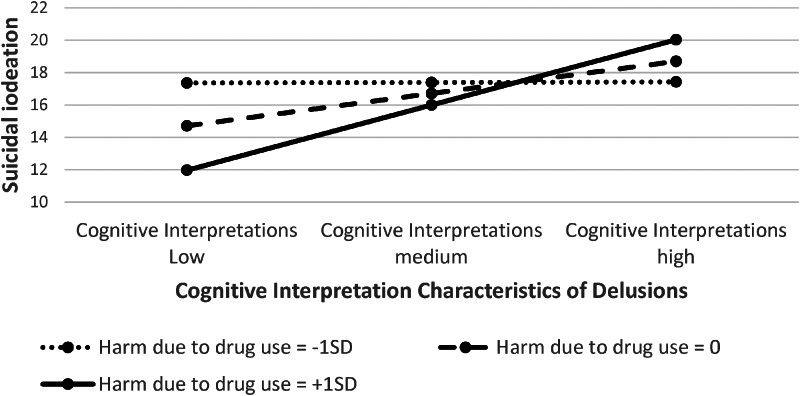
Moderating effect of perceived harm due to drug use on the strength of the relationship between the severity of cognitive interpretation characteristics of delusions and suicidal ideation. This relationship was strongest when harm due to drug use was high (1 s.d. above the mean) and weakest when harm due to drug use was low (1 s.d. below the mean). The same pattern of findings occurred for the relationship between the severity of emotional characteristics of delusions and suicidal ideation.

However, these main effects with the mediators were qualified by a significant interaction (moderation) effect. Harm due to drug use altered the relationship between (i) PSYRATS cognitive interpretation characteristics of delusions and suicidal ideation, and between (ii) PSYRATS emotional characteristics of delusions and suicidal ideation. These relationships with suicidal ideation were significantly positive only when harm due to drug use was medium or high, specifically, at the level of the mean or 1 s.d. greater than the mean (see Table 3 and Fig. 3).

The indirect effects were significant only when harm due to drug use was at the mean or 1 s.d. above the mean. Two pairwise contrasts between the conditional indirect effects were significant: the effect when harm due to drug use was at its highest level *v.* when it was at its lowest level, and the effect when harm due to drug use was at its highest level *v.* when harm due to drug use was at the mean (see Table 3).

In model 3 (*N* = 132) which tested a pathway from PANSS positive symptoms to PSYRATS emotional characteristics of delusions via PSYRATS cognitive interpretations, cognitive interpretations, and emotional characteristics of delusions were positively associated. There was no significant interaction effect meaning that harm due to drug use did not affect the strength of that relationship.

*Harm due to drug use as a moderator with PSYRATS cognitive interpretation and emotional characteristics of auditory hallucinations as mediators* (*N* *=* *125*)

PANSS positive symptoms of psychosis were positively associated with the mediators of PSYRATS cognitive interpretations and emotional characteristics of auditory hallucinations and there was no significant moderation effect of harm due to drug use, similar to the previous analysis with delusions. There was also no significant association between PANSS positive symptoms and suicidal ideation. However, unlike the findings with delusions, harm due to drug use did not affect the relationships between the two auditory hallucination mediators and suicidal ideation. Neither were there any significant interaction effects in model 3 (*N* = 128).

*Harm due to alcohol use as a moderator with PSYRATS cognitive interpretation and emotional characteristics as mediators of delusions* (*N* *=* *168, 169*) *and auditory hallucinations* (*N* *=* *165, 169*)

When harm due to alcohol use was a moderator, there were no significant interaction effects involving the two PSYRATS cognitive interpretation and emotional mediators for either delusions or auditory hallucinations. For model 3, consistent with the previous analyses, harm due to alcohol use did not affect the relationship between cognitive interpretations and emotional characteristics for either delusions or auditory hallucinations.

**Research Question 3: Which of the components of suicidal ideation were most affected by moderated mediated pathways?**

The significant moderated mediation models with harm due to drug use as a moderator and the two mediators of PSYRATS cognitive interpretation and emotional characteristics of delusions was applied to each of the five components of suicidal ideation, namely, intensity, active desire, planning, passive attempt, and lack of deterrents. There were significant interaction (moderation) effects with cognitive interpretation characteristics (*N* = 115), but not emotional characteristics, for three of the five components, namely, active desire, passive attempts, and lack of deterrents. The interaction effects with intensity and planning just failed to reach significance.

## Discussion

This is the first study to examine the role of harm due to substance use in pathways to suicidal ideation comprising not only the presence of positive psychotic symptoms but the severity of the cognitive and emotional sequelae of such symptoms. There were three novel findings.

First, there was evidence of an indirect sequential pathway linking PANSS positive psychotic symptoms and suicidal ideation with two mediators of (i) cognitive interpretation characteristics and (ii) emotional characteristics of both delusions and auditory hallucinations as measured by the PSYRATS. Although evidence of simple binary relationships between positive psychotic symptoms and suicidal thoughts/acts is relatively robust (Hawton, Sutton, Haw, Sinclair, & Deeks, 2005; Olfson et al., 2021; Qin, 2011) and also replicated in the current study, the influence of the experienced cognitive and emotional characteristics of auditory hallucinations and delusions on suicidal thoughts/acts has been rarely investigated. That said, simple associations between the emotional characteristics of auditory hallucinations and suicidal ideation in Malaysian psychiatric inpatients with schizophrenia have been previously documented (Chong et al., 2020). In addition, evidence was published for a pathway in which the emotional characteristics of auditory hallucinations led to suicidal thoughts and behaviors via a mediator of depression, again in psychiatric inpatients with schizophrenia recruited from a large Chinese hospital (Yin et al., 2023). Our findings advance the understanding of these types of pathways by showing that (i) *both* the cognitive interpretation and emotional domains of auditory hallucinations *and* delusions were key in pathways to suicidal thoughts, and (ii) cognitive interpretations of auditory hallucinations and delusions preceded emotional characteristics but not the other way round. While it is intuitively compelling that feeling controlled by voices or feeling preoccupied by delusional thoughts might lead to emotional distress in pathways to suicidal experiences, this has not been tested previously. This finding underscores the importance for future work to go beyond charting the presence of positive psychotic symptoms in pathways to suicidal thoughts and to comprehensively investigate the perceived cognitive and emotional consequences of different aspects of those experiences.

The second novel finding extended the first by showing that self-perceived harm due to drug use moderated associations between cognitive interpretations and emotional characteristics of delusions and suicidal ideation. The indirect effect from PANSS psychotic symptoms to suicidal ideation via these two mediators was significant only when harm due to drug use was at medium or high levels. Hence, the moderated mediation model depicted in Fig. 1 was supported but only for delusions and not for auditory hallucinations. Furthermore, and in contrast to the effects of harm due to drug use, harm due to alcohol use did not act as a moderator in these pathways. Previous work which has examined moderating effects of substance use on relationships involving suicidal thoughts/acts is comparatively sparse. One exception was a study in which the frequency of using 12 substances (including alcohol) affected suicidal thoughts via feelings of being a burden, and suicide attempts via having the means to make an attempt (Baer, Tull, & Gratz, 2022). Micro-longitudinal diary work used together with case studies and in-depth qualitative interviews are now needed to comprehensively probe the complex fluctuations in, and interactions between, harm due to substance use in relation to the cognitive and emotional effects of living with auditory hallucinations, delusions, and suicidal experiences.

The third novel finding was that when the five different components of suicidal ideation were investigated, namely, intensity, planning, active desire, passive desire, and lack of deterrents (Beck & Steer, 1991), the last three components were affected most strongly and via the pathway involving the moderating effect of harm due to drug use on cognitive interpretations of delusions. A passive desire to die represents feelings of not wanting to exist anymore but not necessarily to die by suicide. An example is that in people who use street drugs, thoughts of passive suicide may manifest as ‘hope’ for an accidental overdose. Passive suicidal desires are often overlooked clinically and, in general, are under-researched. This third finding highlights the importance of taking a fine-grained approach to understanding fluctuations within and between different types of suicidal experiences, perhaps using convergent diary and qualitative methods.

There were four key limitations of this study. First, the design was cross-sectional meaning that strong conclusions cannot be made about temporal precedence. Second, discerning the effects of different types of substances (e.g. cannabis, opioids, spirits) on relationships between psychotic experiences and suicidal thoughts/acts represented a level of complexity beyond the scope of the current study. Third, harm due to substance use is multi-componential encompassing, for example, alienation of family, criminal activity, spiraling debt, and physical health problems, the detailed effects of which could not be extricated by the current work. Fourth, auditory hallucinations were a focus. Future work might benefit from examining any differential effects of the different modalities in which hallucinations can manifest.

Four strengths should also be noted. First, participants had acute psychotic and suicidal experiences and were recruited from a range of mental health services including psychiatric inpatient wards and community teams (e.g. early intervention, home treatment, and crisis teams). These sample characteristics are rare. Second, it was *self-perceived* harm due to substance use which was key. This is important because self-perceived harm presents a potentially acceptable starting point for effecting change. Third, we were able to statistically account for general effects of having a ‘severe mental illness’, for example, depression and/or anxiety. Third, five components of suicidal ideation were investigated. This is important not only for gaining a better understanding of the mechanisms underlying suicidal experiences but also for the development and application of suicide focused interventions clinically (Gooding et al., 2020; Tarrier et al., 2013). Future intervention development needs to not only be formulation-based and explicitly suicide-focused but also to address effects of therapy on different domains of suicidal experiences.

In conclusion, this study has important and overlapping scientific and clinical implications. It is vital to develop a more in-depth understanding of the interacting complexities arising from harmful substance use; the cognitive and emotional impacts of hallucinations and delusions; and different types of suicidal experiences. Mental health services sometimes attempt to separate the needs of individuals who use substances from needs reflecting psychotic and/or suicidal experiences in those same individuals despite contrary recommendations. It has been argued that a more integrated approach is necessary (Moriarty, 2020). Furthermore, mental health provision tends not to have an explicit focus on suicidal thoughts and acts, which should be redressed (Gooding et al., 2020). To take this further, it is clear that mental health services should effectively implement therapeutic provision which addresses multifaceted mental health problems comprising any degree of substance use, positive psychotic symptoms, and any suicidal experiences.
